# Introduction to a New Open Access Journal by MDPI: *Epidemiologia*

**DOI:** 10.3390/epidemiologia1010001

**Published:** 2020-08-06

**Authors:** Antoine Flahault

**Affiliations:** Institute of Global Health, Faculty of Medicine, University of Geneva, CH-1202, 8001 Geneva, Switzerland; Antoine.Flahault@unige.ch

I am pleased to announce a new journal in the field of epidemiology. *Epidemiologia* will provide a platform for scientists and academics all over the world to promote, share, and discuss various new issues and developments in the field of epidemiology. 

The first phase of the journal is focusing on expanding the editorial board, so as to embrace top epidemiologists from all over the world and publish high quality papers. I would like to take this opportunity to thank current editorial members for their commitment to the standard to which *Epidemiologia* aspires. 

*Epidemiologia* will publish papers covering a broad range of areas, including but not limited to: field epidemiology; theoretical epidemiology; cancer epidemiology; epidemiology of communicable diseases; epidemiology of non-communicable diseases; epidemiology of mental health; environmental and planetary epidemiology; infectious disease epidemiology; nutritional epidemiology; occupational injury and illness epidemiology; clinical epidemiology; molecular epidemiology, etc.

The open access of the journal will provide maximal dissemination of the published research, reaching an almost unlimited readership. In this way, the large and heterogeneous scientific community interested in epidemiology will be reached without any restrictions. Accordingly, the access will allow the journal to optimally serve its function, i.e., the promotion of research by distributing pertinent experimental knowledge and scientific ideas.

We welcome you to *Epidemiologia*, and invite you to contribute your papers or submit special issue proposals. We look forward to receiving your manuscripts for publication in this journal.

